# Robert Spitzer's legacy: agreement is halfway to truth

**DOI:** 10.1192/bjb.2018.22

**Published:** 2018-10

**Authors:** Peter Tyrer

**Affiliations:** 1 Imperial College London, UK

## Abstract

Bob Spitzer died in December 2015 but is not likely to be forgotten quickly. His Herculean task in reforming psychiatric diagnosis, mainly by the development of the third edition of the *Diagnostic and Statistical Manual of Mental Disorders* in 1980, has been acknowledged by many, but it remains unfinished. To complete the process, we need more than good diagnostic reliability, we need better veracity.

**Declaration of interest:**

None.

Most psychiatrists will have heard of Robert Spitzer, but outside the USA he is essentially known for his association with DSM-III, the defining third edition of the *Diagnostic and Statistical Manual of Mental Disorders*.^1^ Even this association took some time to establish, and in my lectures I used to explain that the rather unusual name of Spitzer was only an acronym for ‘Structured Psychiatric Interviews To Zealously Enhance Research’, until people started to believe he was not a real person. Then I stopped.

Shortly after this, I was giving a lecture on the classification of personality disorder in the USA. It was a fairly light-hearted event, and I presented the talk as a debate between two protagonists, one supporting a categorical classification and the other a dimensional one. When I was criticising borderline personality disorder from the dimensional perspective, I joked, ‘the classification of borderline personality disorder in DSM-III was only approved after a dead heat on the vote, so it was decided to include it by the casting vote of the chairman. Is this the way to organise a classification system?’

A voice growled from the audience: ‘What's the matter with you? Don't you believe in democracy?’ The voice came from Robert Spitzer. I have thought about this event continually since and concluded that the single word that summarises the work of Robert Spitzer is ‘agreement’. This needs explanation.

Before 1980, international psychiatric classification was in a mess. There can be no other word for it. There were large national differences in the incidence of major mental disorders such as schizophrenia,^2^ fractured understanding because of conflict between psychodynamic and organic schools, and a general cynical attitude towards diagnosis by clinicians, who essentially picked a treatment first and gave the nearest diagnosis afterwards.

Robert (Bob) Spitzer changed all this with his approach to DSM-III. He, and the American Psychiatric Association, recognised that, without substantial agreement in diagnostic assessment, psychiatry was going nowhere. Robert had developed a system using operational criteria to improve diagnosis in research, and now he was being asked to extend this to clinical practice. This was a much taller order than might be thought. Clinicians in busy practice do not take kindly to research-based formats, and operational criteria had to be understood by everybody if they were going to be accepted.

Once a rough set of criteria had been developed, it was necessary to test them in practice. Nowadays, this would be carried out in the form of field trials; Bob did not have the time, or the resources, for these. So he organised a set of mass colloquia. All the experts in each given field were invited to meet with him at the New York Psychiatric Institute to sort out the new diagnostic prototypes. In this task, Bob became the Great Persuader. As was noted by David Shaffer, one of the experts brought in to examine diagnoses in child psychiatry, these occasions were fairly chaotic, but Bob was clearly in charge and was found everywhere arguing about detail; a key element of all these meetings was that they continued until agreement was reached on all essentials.^3^ Sometimes, fatigue became the defining factor in getting this agreement; it can be a most effective enabler.

Within days of each meeting, Bob had produced a summary of each meeting in which a new classification emerged with all the levels of agreement listed. A few people scratched their heads – ‘did I really agree to that’ – but they were persuaded by the stellar company of other experts that this was indeed the right way forward. So, when Bob sent in his list of revolutionary proposals to the American Psychiatric Association, he had all the authorities in each field signed up to the new system, and so it had to be accepted.

Views about the DSM-III since its publication in 1980 have oscillated widely. The system of operational criteria worked well for some diagnoses, but not for others such as personality disorder,^4^^,^^5^ and there is continued argument over the status of the portmanteau diagnosis of ‘major depressive disorder’, which covers too wide a range of pathology.^6^ Although Spitzer is credited with the removal of homosexuality from psychiatric diagnosis in the 1970's, it still appeared in DSM-III. There has also been criticism of the expansion of diagnoses and suggestions, some warranted but others unfair, that some of the new diagnoses were engineered to suit the products of pharmaceutical companies.

With each successive version of DSM, the contribution of Bob Spitzer has receded and the volume of dissent has increased, perhaps illustrated most forcefully in the popular writings of Allen Frances.^7^ But Bob kept an eye on these DSM revisions and was far from happy with many of them, particularly when they deviated from his firm notions about agreement, which he was not slow to express.^8^

Of course, agreement is not everything, and the search for validity has to follow. Since DSM-III was published 38 years ago, we have made only the slightest furtive steps towards the aim of independently verified psychiatric diagnoses. Bob Spitzer showed the way, quite brilliantly, but he left us halfway down the track, and it is now up to others to finish the race.

## About the author

**Peter Tyrer** is Emeritus Professor of Community Psychiatry at the Centre for Psychiatry, Imperial College London, UK.
